# The Football Association Injury and Illness Surveillance Study: The Incidence, Burden and Severity of Injuries and Illness in Men’s and Women’s International Football

**DOI:** 10.1007/s40279-020-01411-8

**Published:** 2020-12-28

**Authors:** Bradley Sprouse, Jon Alty, Steve Kemp, Charlotte Cowie, Ritan Mehta, Alicia Tang, John Morris, Simon Cooper, Ian Varley

**Affiliations:** 1 Sport Science Department, Nottingham Trent University, Nottingham, UK; 2 The Football Association, Burton-Upon-Trent, UK

## Abstract

**Objectives:**

To determine the incidence and characteristics of injury and illness in English men’s and women’s senior and youth international football.

**Methods:**

Time-loss injuries and illnesses, alongside match and training exposure, were collected across 8 seasons (2012–2020) in youth (U15, U16, U17, U18, U19) and senior (U20, U21, U23, senior) English men’s and women’s international teams. Analysis of incidence, burden, and severity of injury and illness was completed. Sex-specific comparisons were made between the senior and youth groups, and across the 8 seasons of data collection.

**Results:**

In men’s international football, 535 injuries were recorded (216 senior; 319 youth) during 73,326 h of exposure. Overall, match injury incidence (31.1 ± 10.8 injuries/1000 h) and burden (454.0 ± 195.9 d absent/1000 h) were greater than training injury incidence (4.0 ± 1.0 injuries/1000 h) and burden (51.0 ± 21.8 d absent/1000 h) (both *P* < 0.001). In women’s international football, 503 injuries were recorded (senior: 177; youth: 326) during 80,766 h of exposure and match injury incidence (27.6 ± 11.3 injuries/1000 h) and burden (506.7 ± 350.2 days absent/1000 h) were greater than training injury incidence (5.1 ± 1.8 injuries/1000 h) and burden (87.6 ± 32.8 days absent/1000 h) (both *P* < 0.001). In women’s international football, a group × season interaction was observed for training injury incidence (*P* = 0.021), with the senior group recording a greater training injury incidence during the 2015–2016 season compared to the youth group (14.4 vs 5.7 injuries/1000 h; *P* = 0.022). There was no difference in injury severity between match and training for men’s (*P* = 0.965) and women’s (*P* = 0.064) international football.

**Conclusions:**

The findings provide a comprehensive examination of injury and illness in English men’s and women’s senior and youth international football. Practitioners will be able to benchmark their team’s injury and illness incidence and characteristics to the match-play and training information provided in the present study.

## Key Points

**Table:** 

The incidence and burden of injury were higher in matches, compared to training, in male and female international football.
There was no difference in injury incidence or burden between the senior and youth groups in male or female international football.
The data regarding match and training injury epidemiology in international football will enable practitioners to benchmark their team’s injury and illness characteristics.

## Introduction

Football is a high-intensity intermittent team sport that requires players to be physically capable of coping with high-intensity movements, including repeated changes of direction, sprints, and accelerations/decelerations, alongside complex and physically demanding technical actions [1, 2]. Due to the nature of these activities, it is unsurprising that the incidence of injury and illness in professional football is high [3]. In international football, players may be at an increased risk of injury and illness due to the potentially unaccustomed nature of training and match-play compared to club football [4] and increased fixture congestion [5, 6]. A decrease in player availability as a result of injuries and illnesses has been shown to have a detrimental effect on team performance, and can cause economic adversity to clubs, international football organisations, and governing bodies [7, 8]. In addition, player welfare is of paramount importance, and therefore, appropriate measures should be undertaken to minimise player risk of injury and illness.

Before any attempt is made to introduce injury and illness preventative measures or interventions, it is important to understand the epidemiology of injuries and illnesses (incidence, burden, severity, cause, and onset) in the population of interest [9]. In men’s club football, the UEFA Elite Club Study (UECS) [10] has provided a commonality in definitions and the injury and illness reporting process [11], and has provided an insight into injury and illness in men’s professional football in the European leagues [10, 12]. Epidemiological information also exists in elite youth football [13, 14]. However, while these studies provide useful information, data from international or women’s football does not form part of their analyses.

In a recent meta-analysis, injury incidence was shown to be higher in men’s international tournament football compared to professional club football (9.8 vs 7.5 injuries per 1000 h; [15]). This suggests that injuries are of particular concern at the international level, and therefore, developing a greater understanding is imperative to aid practitioners in the monitoring and prevention of injuries in international football. The majority of the published literature concerning injury in men’s international football is based around senior players in tournament match-play [15]. While these data provide an insight into the incidence of injury during tournaments (33.9–48.2 injuries per 1000 h [15]), longitudinal data are scarce concerning all elements of international football (e.g., friendly matches, training camps). International teams are likely to meet for training camps and qualifying fixtures > 6 times per year (50–80 days for senior/U21 teams), which could equate to a large proportion (20–25%) of their training and match-play load. Information on the epidemiology of injury and illness across all international football activity (i.e., training camps, friendly fixtures, competitive fixtures, and tournaments) is required to provide a greater understanding of the injury and illness risk of playing international football.

The popularity of women’s football is increasing. In England alone, the number of affiliated women’s and girls’ teams has increased from 6000 in 2017 to over 12,500 teams in 2020 [16]. The professional game also continues to grow, as evidenced by UEFA reporting a > 200% increase in professional players between 2012/13 and 2016/17 [17]. As the popularity of women’s football increases, there is a requirement to understand the likely increased impact of football on injury and illness in elite female players. There are a distinct lack of published research studies documenting the epidemiology of injuries in elite women’s football. The studies that do exist often include a low number of injuries (< 100; [18, 19]), which limits the conclusions that can be drawn, and are conducted in amateur players [20–22], who are likely to have a wide range of physical capabilities and training exposure levels, and therefore may suffer from a different pathophysiology of injury in comparison to professional players [23].

Studies assessing elite female  players from the leading leagues in Europe and North America provide an insight into injury incidence in club-based match-play (12.6–22.6 injuries per 1000 match hours) and training (1.2–3.8 injuries per 1000 training hours) [24–27]. However, information regarding injury incidence in women’s elite international football is scarce. Previous studies in women’s international football have focused only on international tournaments, rather than international football as a whole [28, 29]. Data from tournaments staged between 1999 and 2006, involving under 19 through to Senior players, showed that the match injury incidence ranged from 20 to 49 injuries per 1000 h [28], while an average of 1 time-loss injury per match was reported in FIFA senior World Cups between 1999 and 2011 [28]. Injury information voluntarily collected from teams competing in international tournaments has previously been provided following each game. The competitive nature of international football may have led to some omissions in the data provided by some teams and may be an underrepresentation of the injury incidence rate of international tournament football. The lack of information regarding injury incidence in women’s international football outside of a tournament setting and the underreporting of other injury metrics, such as burden and severity, suggests that the data available so far are insufficient to assess the injury risk of women’s international football. Consequently, several important questions relating to the epidemiology of injuries in women’s international football remain unanswered.

Due to numerous factors, including increased physicality of football participation associated with increasing age [30] and maturation status [31], injury epidemiology in senior football may be different to youth football [32]. However, previous research predominantly focusses on senior international or club football, with a scarcity of longitudinal research in international youth football, and therefore, it is difficult to determine if differences occur. The provision of such information would not only be of great interest to applied practitioners, but also act as a benchmark to enable the success of any future injury preventative measures implemented in an international setting.

Therefore, aims of the current study were to, first, determine the incidence, burden, and severity of injury and illness during training and match-play in elite men’s and women’s senior and youth international football across 8 seasons (2012–2020). Due to the established anthropometric and physiological differences between male and female footballers [33, 34], a comparison of injury and illness metrics between men and women was not the aim, and is beyond the scope, of the current study. This paper will however address sex-specific comparisons in injury incidence, burden, and severity between senior and youth age groups in international football.

## Method

### Design

The English Football Association (FA) is keen to promote the undertaking of good medical practice standards throughout professional football, including in men’s and women’s club football and in the men’s and women’s England national teams. To realise the aim of acquiring World-leading status in the treatment and prevention of injury and illness in football, the FA introduced the Injury and Illness Surveillance Study in 2012. Since this date, the study has grown to incorporate > 50 teams in both men’s and women’s professional football and the national teams. The present study represents a subset of the data collected as part of this study.

### Participants

The data presented in this study comprise men’s (Under 15, 16, 17, 18, 19, 20, 21, Senior) and women’s [Under 15, 16, 17, 18, 19, 20, 21, 23, Senior (Under 20 2014–2019; Under 21 2018–2019; Under 23 2012–2019)] data from 8 international seasons. To ensure clarity of reporting, for the purposes of the current study, the age groups have been separated into youth (Under 15, 16, 17, 18, 19) and senior (Under 20, 21, 23, Senior) groups. The group characteristics for the study period are shown in Table 1.

**Table 1:** Sample characteristics of the men’s and women’s senior and youth groups used in the present study

	Age group	Season								
		12/13	13/14	14/15	15/16	16/17	17/18	18/19	19/20	Total
Men										
Number of camps	U15–U19	15	30	27	29	32	30	25	20	208
	U20–Senior	14	12	17	14	17	14	16	9	113
Number of Matches	U15–U19	30	51	57	68	73	72	54	48	453
	U20–Senior	24	28	36	34	39	36	29	18	244
Match exposure (h)	U15–U19	458	752	875	1022	1159	1139	849	760	7014
	U20–Senior	396	453	583	538	644	594	490	302	4000
Training exposure (h)	U15–U19	1880	3864	3514	4345	6903	6197	5935	4112	36,750
	U20–Senior	1981	2025	3230	2222	3763	5749	4408	2186	25,564
Camp days	U15–U19	123	207	240	294	298	282	268	171	1883
	U20–Senior	115	125	221	158	171	186	223	98	1297
Number of players	U15–U19	277	639	554	626	631	601	551	453	4332
	U20–Senior	316	346	456	295	343	373	398	216	2743
Number of Injuries	U15–U19	20	45	46	43	63	47	31	24	319
	U20–Senior	38	21	37	14	30	30	35	11	216
Women										
Number of camps	U15–U19	17	25	30	36	39	40	40	27	254
	U20–Senior	11	15	15	11	21	23	15	7	118
Number of Matches	U15–U19	26	38	36	41	48	58	37	31	315
	U20–Senior	20	19	32	15	21	27	36	13	183
Match exposure (h)	U15–U19	405	606	548	636	788	919	570	517	4989
	U20–Senior	330	315	540	248	347	446	594	218	3038
Training exposure (h)	U15–U19	3696	5395	5918	5749	7906	7387	4979	5132	46,162
	U20–Senior	1966	2814	3373	1589	4316	5573	5009	1939	26,579
Camp days	U15–U19	101	178	173	185	263	238	254	148	1540
	U20–Senior	79	103	160	84	149	189	192	73	1029
Number of players	U15–U19	484	710	732	844	893	958	606	625	5852
	U20–Senior	242	336	351	248	440	524	309	142	2592
Number of Injuries	U15-U19	24	33	33	59	31	51	50	45	326
	U20-Senior	12	23	25	23	14	30	39	11	177

Informed consent from all players was obtained prior to data collection, and participants were informed that they could withdraw from the study at any time without consequence. Ethical approval was granted by the Nottingham Trent University Non-Invasive Ethical Review Committee (application number: 116V2). The study was performed in accordance with the standards of ethics outlined in the Declaration of Helsinki. The sample size was determined by the nature of the group, in that the number of players selected for the international squads and, subsequently, the participants that agreed for their data to be used in the study, determined the number of participants. In total, the present study includes 7075 player camp attendances across the 8 different male squads over 8 years of data collection and 8367 player camp attendances across 9 different female playing squads over 8 years of data collection.

### Data Collection

Data were collected and analysed based on the international consensus statement on the process of conducting epidemiological studies in professional football [35]. Amendments were made when recording severity, where injuries greater than 28 days but less than 90 days were categorised as severe, and injuries greater than 90 days were categorised as major. In line with the recent International Olympic Committee Consensus Statement [36], injuries of 7 days or less were categorised as minor (Table 2). Amendments were also made when recording cause of injury, where cumulative injuries were reported as well as contact and non-contact injuries, given that cumulative injuries are frequently reported in applied practice.

**Table 2:** Injury classification headings and options

Column heading	Description
Event	Match/training/non-club related/other
Date of injury and return date	Estimated return date provided if player had not returned to play inside the data collection period. *followed up with club for actual return date
Injury Onset	Acute/gradual
Injury Cause	Contact/non-contact/cumulative

Data on injury and illness incidence were collected for the entirety of all international camps, in each age group, from 2012 to 2020. Any injuries and illnesses that occurred during the international camps were included in the data analysis. Before the study commenced, all medical support staff for each age group were provided with guidance on why the study was taking place, and information on the definitions of each variable that required input. A mailbox was also created to allow for questions to be raised by individual practitioners. Injuries that lasted longer than the camp length were followed up with clubs to gain accurate return dates (for the purpose of severity calculations).

An injury was defined as an occurrence sustained in a national team-related activity (training or match-play) which prevented a player from taking part in training or match-play for 1 or more days following the occurrence [35]. Illness was defined as a medical condition which prevented a player from taking part in training or match-play for 1 or more days following the illness [35].

Injury and illness information was recorded and classified [37] by each team’s medical support staff using their electronic medical record system (PMA, The Sports Office, Wigan, United Kingdom). The information collected for injuries is shown in Table 2. Training and match exposure data were collected via a bespoke exposure collection form. Guidance documents were provided on how to complete and record the information.

As a minimum, teams were asked to provide descriptive information related to onset (acute/gradual) and cause of injury (contact/non-contact/cumulative), as shown in Table 2**.** Injuries sustained outside of formal training and match-play were excluded from the analyses. Teams were also asked to provide descriptive information for illness including date of reporting and return date (indicating when a player was available to return to training or match-play). The protocol for recording illnesses was the same as injuries.

The severity of injuries and illnesses was defined by the number of days that the player was unable to take part in regular training or match-play. To categorise injury severity, the following thresholds were adhered to: 1–7 days absence (minor), 8–28 days (moderate), 29–89 days (severe), and ≥ 90 days (major); based on previous research [35, 36].

### Exposure

Any formal match-play (international fixtures against another country) and all training time that took place during an international camp was recorded by team support staff (Sports Scientists, Physical Performance Coaches) to form a match and training exposure value, as follows:

*Match Exposure* the total minutes played by each player during a match.

*Training Exposure* reported on a squad basis with modification information provided for players that were not involved in the ‘regular’ training session. Player leisure activities outside of structured training and match-play were not recorded.

### Data Analysis

Mean values were reported ± SD. Identical analyses were performed separately on the men’s and women’s datasets. Incidence and burden were calculated via the following formulae:

$$\begin{gathered} {\text{Incidence}} = \left( {{\text{total number of injuries}} \times {1}000} \right)/{\text{total exposure}}\;{\text{h}} \hfill \\ {\text{Burden = }}\left( {{\text{total number of days absent}} \times {1}000} \right)/{\text{total exposure}}\;{\text{h}}{.} \hfill \\ \end{gathered}$$

A 2-way (event × season) ANOVA was used to compare differences in match and training injury incidence and burden overall, and for both senior and youth groups. A 2-way (group × season) ANOVA was used to compare sex-specific seasonal differences between senior and youth groups for incidence and burden of injury across the 8 seasons. Where significant interactions were observed, post hoc pairwise comparisons were performed using a Bonferroni correction. The Chi-square (*χ*^2^) test of independence was used to determine differences in the distribution of injuries between the senior and youth groups for injury severity (minor, moderate, severe, major), onset of injury (acute, gradual), and cause of injury (contact, non-contact, cumulative). Independent sample *t* tests were used to determine differences in illness incidence and burden between senior and youth groups. All statistical analyses were completed in SPSS (IBM SPSS Statistics, Version 24, 2018) and Microsoft Excel (Microsoft Windows 10, 2018), with statistical significance set at *P* < 0.05.

## Results

### International Men’s Football

#### Exposure

Total exposure over the duration of the study period was 73,326 h (senior: 29,562 h; youth: 43,764 h) consisting of 11,010 match hours (senior: 3998 h; youth: 7012 h) and 62,316 training hours (senior: 25,564 h; youth: 36,752 h). There were 24 ± 3 (senior) and 21 ± 1 (youth) players involved in each camp and 14 ± 3 senior and 26 ± 6 youth camps per season; resulting in 162 ± 47 senior and 235 ± 63 youth camp days per season. There were 31 ± 7 (senior) and 57 ± 14 (youth) matches per season, resulting in 500 ± 114 (senior) and 877 ± 231 (youth) h of match exposure. In total, there were 3196 ± 1369 (senior) and 4594 ± 1650 (youth) h of training exposure per season.

#### Injury Incidence

**Fig. 1:**
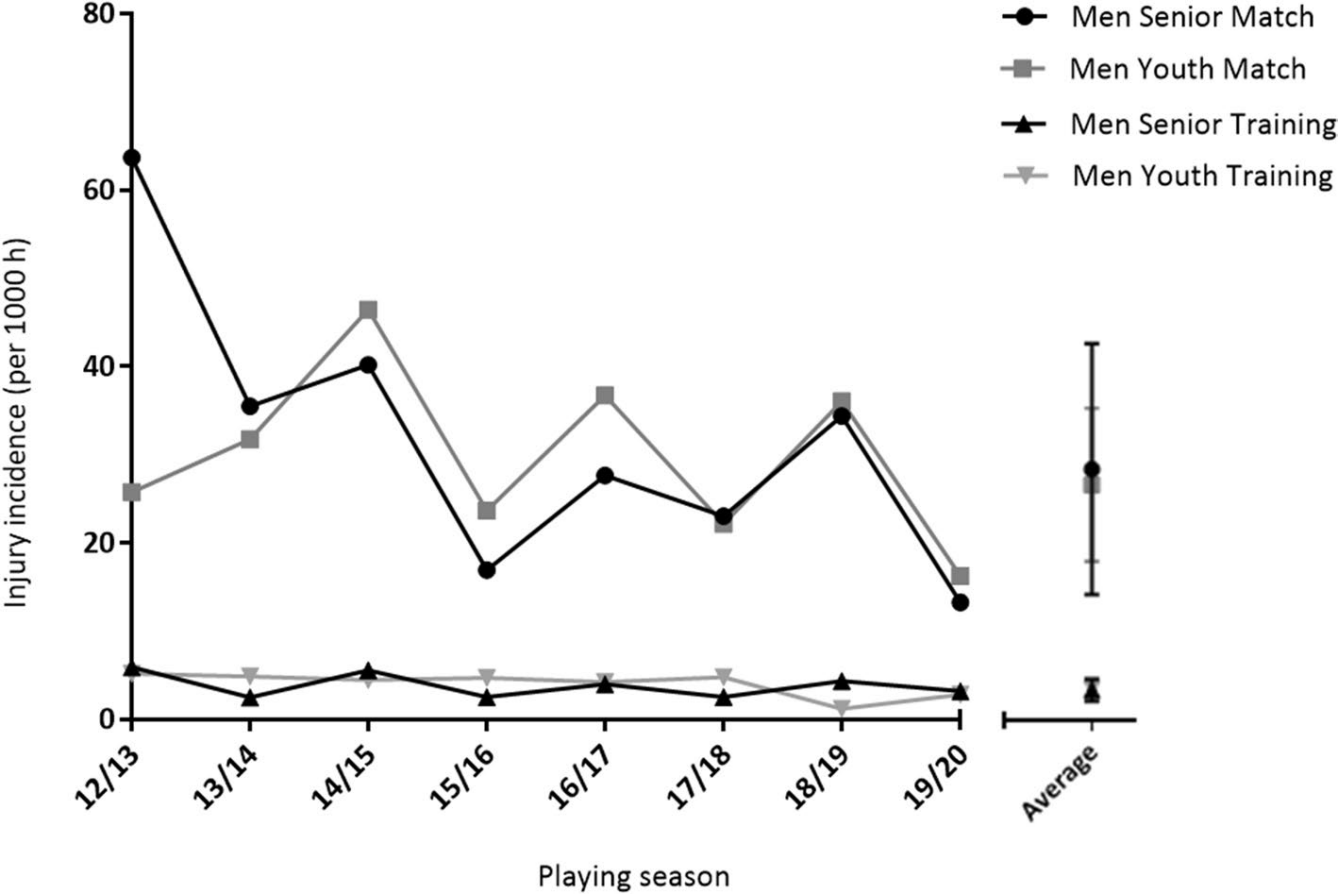
Injury incidence (per 1000 h) for men’s senior and youth international teams from 2012 to 2020

There were a total of 535 injuries recorded (216 senior; 319 youth) across 8 seasons. These consisted of 312 match injuries (126 senior, 186 youth) and 223 training injuries (90 senior, 133 youth). Match injury incidence was 31.8 ± 15.9 injuries per 1000 h (senior) and 29.8 ± 9.7 injuries per 1000 h (youth). Training injury incidence was 3.8 ± 1.4 injuries per 1000 h (senior) and 4.0 ± 1.4 injuries per 1000 h (youth) (Fig. 1).

Overall match injury incidence (31.1 ± 10.8 injuries per 1000 h) was higher in comparison to training injury incidence (4.0 ± 1.0 injuries per 1000 h) (main effect of event, *P* < 0.001), with no difference observed over 8 seasons (main effect of season, *P* = 0.125) and no event × season interaction (*P* = 0.230). Match injury incidence was higher than training injury incidence for the senior group (31.8 ± 15.9 vs. 3.8 ± 1.4 injuries per 1000 h; main effect of event, *P* < 0.001). There was also a main effect of season (*P* = 0.037), with post hoc analyses revealing that injury incidence was higher in the 2012–2013 season compared to the 2019–20 season (*P* = 0.041). However, no event × season interaction was observed (*P* = 0.104). Match injury incidence was higher than training injury incidence for the youth group (29.8 ± 9.7 vs. 4.0 ± 1.4 injuries per 1000 h; main effect of event, *P* < 0.001); however, no difference was observed over 8 seasons (main effect of season, *P* = 0.742) with no event × season interaction (*P* = 0.732).

There was no significant difference in injury incidence between the youth and senior groups (main effect of group: match *P* = 0.770; training *P* = 0.808) or over 8 seasons (main effect of season: match *P* = 0.260; training *P* = 0.754), with no group × season interaction (match *P* = 0.774, training *P* = 0.695).

#### Injury Burden

**Fig. 2:**
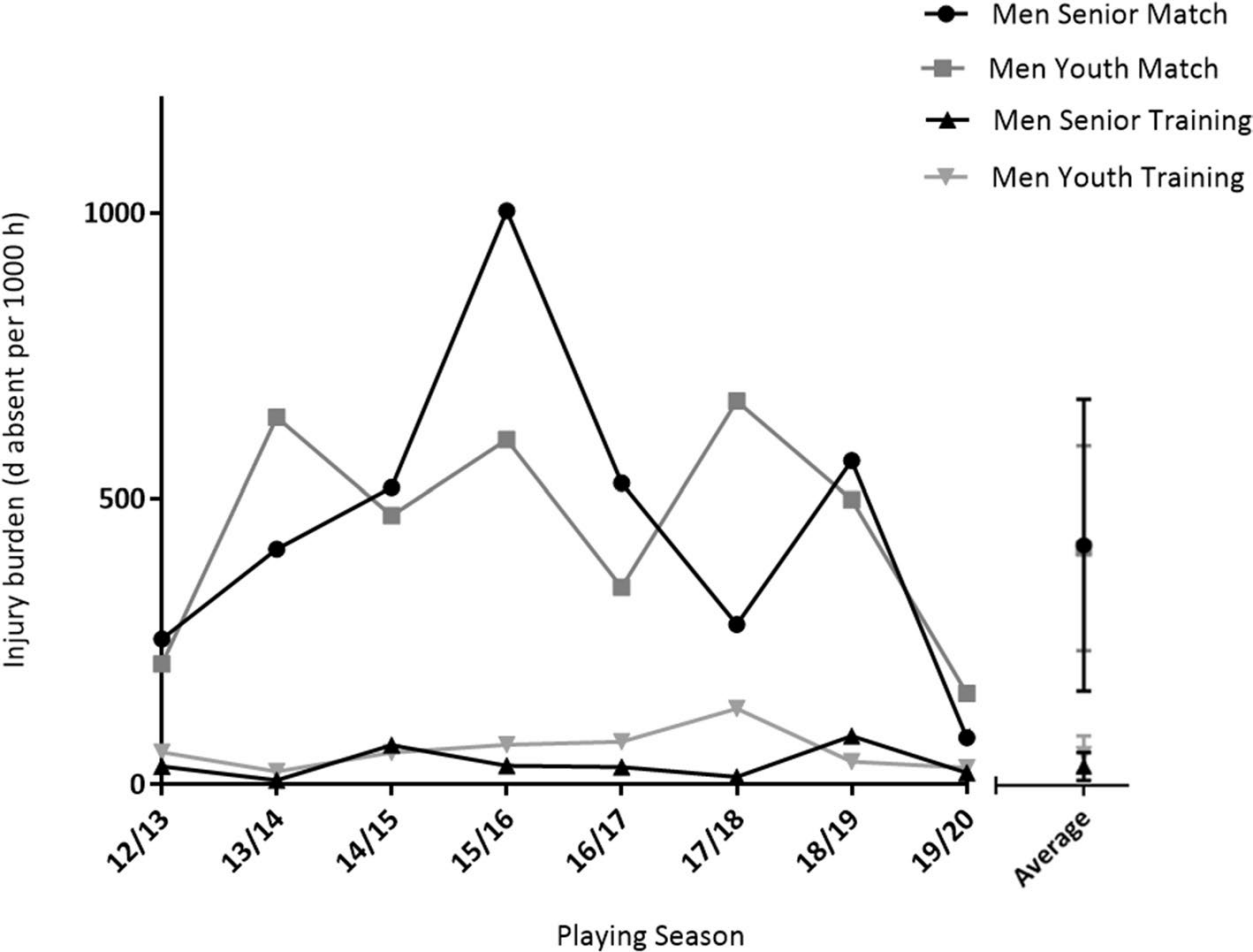
Injury burden (days absent per 1000 h) for men’s senior and youth international teams from 2012 to 2020

The match injury burden of international football was 455.7 ± 276.4 days absent per 1000 h (senior) and 450.0 ± 194.6 (youth) days absent per 1000 h, while the injury burden as a result of training was 36.2 ± 27.0 (senior) and 60.2 ± 34.6 (youth) days absent per 1000 h (Fig. 2).

Overall match injury burden (454.0 ± 195.9 days absent per 1000 h) was higher in comparison to training injury burden (51.0 ± 21.8 days absent per 1000 h; main effect of event, *P* < 0.001), with no difference observed over the course of 8 seasons (main effect of season, *P* = 0.349) and no event × season interaction (*P* = 0.466). Match injury burden was higher than training injury burden for the senior group (455.7 ± 276.4 vs. 36.2 ± 27.0 days absent per 1000 h; main effect of event, *P* = 0.001); however, there was no difference observed over the course of 8 seasons (*P* = 0.529) and no event × season interaction (*P* = 0.607). Match injury burden was higher than training injury burden for the youth group (450.0 ± 194.6 vs 60.2 ± 34.6 days absent per 1000 h; main effect of event, *P* < 0.001); however, no difference was observed over the course of 8 seasons (main effect of season, *P* = 0.750) with no event × season interaction (*P* = 0.867).

There was no difference in injury burden between the youth and senior groups as a whole (main effect of group: match *P* = 0.970; training *P* = 0.284) or over the course of 8 seasons (main effect of season: match *P* = 0.456; training *P* = 0.910), with no group × season interaction (match *P* = 0.929, training *P* = 0.731).

#### Injury Severity

There was no difference in the distribution of injuries between match-play and training (*P* = 0.965). No difference in the distribution of injuries between senior and youth groups for overall injury severity (*P* = 0.127), match injury severity (*P* = 0.199), or training injury severity (*P* = 0.306) was shown (Table 3). The incidence and burden for injury severity for the men’s senior and youth groups is shown in Table 4.

**Table 3:** Injury severity, cause, and onset in men’s senior and youth groups (injury number and proportion (%)

	Both groups			Senior			Youth		
	All injuries	Match injuries	Training injuries	All injuries	Match injuries	Training injuries	All injuries	Match injuries	Training injuries
Severity	(*n* = 525)	(*n* = 307)	(*n* = 218)	(*n* = 215)	(*n* = 126)	(*n* = 89)	(*n* = 310)	(*n* = 181)	(*n* = 129)
Minor	314 (60%)	184 (60%)	130 (60%)	130 (60%)	75 (60%)	55 (62%)	184 (59%)	109 (60%)	75 (58%)
Moderate	144 (27%)	84 (27%)	60 (28%)	57 (27%)	32 (25%)	25 (28%)	87 (28%)	52 (29%)	35 (27%)
Severe	53 (10%)	30 (10%)	23 (11%)	26 (12%)	17 (13%)	9 (10%)	27 (9%)	13 (7%)	14 (11%)
Major	14 (3%)	9 (3%)	5 (2%)	2 (1%)	2 (2%)	0 (0%)	12 (4%)	7 (4%)	5 (4%)
Cause	(*n* = 371)	(*n* = 225)	(*n* = 146)	(*n* = 156)	(*n* = 96)	(*n* = 60)	(*n* = 215)	(*n* = 129)	(*n* = 86)
Contact	215 (58%)	150 (67%)^c^	65 (45%)	84 (54%)	60 (63%)	24 (40%)	131 (61%)^a^	90 (70%)	41 (48%)^a^
Non-contact	118 (32%)	56 (25%)	62 (42%)^d^	63 (40%)^b^	29 (30%)	34 (57%)^b^	55 (26%)	27 (21%)	28 (33%)
Cumulative	38 (10%)	19 (8%)	19 (13%)^d^	9 (6%)	7 (7%)	2 (3%)	29 (13%)^a^	12 (9%)	17 (20%)^a^
Onset	(*n* = 462)	(*n* = 276)	(*n* = 186)	(*n* = 187)	(*n* = 114)	(*n* = 73)	(*n* = 275)	(*n* = 162)	(*n* = 113)
Acute	352 (76%)	218 (79%)	134 (72%)	150 (80%)	93 (82%)	57 (78%)	202 (73%)	125 (77%)	77 (68%)
Gradual	110 (24%)	58 (21%)	52 (28%)	37 (20%)	21 (18%)	16 (22%)	73 (27%)	37 (23%)	36 (32%)

NB. The *n* represents the number of injuries for which the severity, cause, and onset were known in each category (10 injuries had an unknown severity, 164 injuries had an unknown cause, and 73 injuries had an unknown onset)

^a^Greater proportion in youth compared to senior

^b^Greater proportion in senior compared to youth

^c^Greater proportion in match-play compared to training

^d^Greater proportion in training compared to match-play (all *P* < 0.05)

**Table 4:** Injury incidence (injuries per 1000 h) and burden (days absent per 1000 h) for severity, cause, and onset in men’s senior and youth groups

	Both groups						Senior						Youth					
	All injuries		Match injuries		Training injuries		All injuries		Match injuries		Training injuries		All injuries		Match injuries		Training injuries	
	Incidence	Burden	Incidence	Burden	Incidence	Burden	Incidence	Burden	Incidence	Burden	Incidence	Burden	Incidence	Burden	Incidence	Burden	Incidence	Burden
Severity																		
Minor	4.3	13.5	16.7	52.9	2.1	6.5	4.4	13.1	18.8	54.3	2.2	6.6	4.2	13.8	15.5	52.1	2.0	6.5
Moderate	2.0	28.5	7.6	109.0	1.0	14.3	1.9	29.0	8.0	120.6	1.0	14.7	2.0	28.2	7.4	102.4	1.0	14.0
Severe	0.7	34.2	2.7	127.3	0.4	17.7	0.9	39.9	4.3	200.6	0.4	14.8	0.6	30.3	1.9	85.6	0.4	19.7
Major	0.2	36.2	0.8	177.5	0.1	11.2	0.1	16.2	0.5	119.8	0.0	0.0	0.3	49.7	1.0	210.3	0.1	19.1
Cause																		
Contact	2.9	45.5	13.6	211.9	1.0	16.1	2.8	41.9	15.0	258.4	0.9	8.1	3.0	47.9	12.8	185.4	1.1	21.7
Non-contact	1.6	32.9	5.1	131.0	1.0	15.6	2.1	29.7	7.3	130.1	1.3	14.0	1.3	35.1	3.9	131.5	0.8	16.7
Cumulative	0.5	6.0	1.7	27.1	0.3	2.3	0.3	1.5	1.8	7.5	0.1	0.5	0.7	9.0	1.7	38.2	0.5	3.5
Onset																		
Acute	4.8	73.6	19.8	325.7	2.2	29.1	5.1	70.1	23.3	379.7	2.2	21.6	4.6	76.0	17.8	294.9	2.1	34.3
Gradual	1.5	22.9	5.3	88.6	0.8	11.3	1.3	11.1	5.3	39.5	0.6	6.7	1.7	30.9	5.3	116.5	1.0	14.5

#### Onset of Injury

There was no difference in the distribution of injuries between match-play and training (*P* = 0.086). There was no difference in the distribution of injuries between senior and youth groups for the overall onset of injury (*P* = 0.094), onset of injuries in matches (*P* = 0.375), or the onset of injuries in training (*P* = 0.140) (Table 3). The incidence and burden for onset of injury for the men’s senior and youth groups is shown in Table 4.

#### Cause of Injury

Contact injuries accounted for a greater proportion of injuries in match-play compared to training, while non-contact and cumulative injuries accounted a greater proportion of training injuries compared to match-play (*P* < 0.001). Contact and cumulative injuries accounted for a higher proportion of injuries in youth players compared to senior players, while non-contact injuries accounted for a higher distribution of injuries in senior players compared to youth players (*P* = 0.002). No difference was observed for cause of injury for match injuries between senior and youth groups (*P* = 0.272). However, contact and cumulative injuries accounted for a higher proportion of training injuries in youth compared to senior players, whereas non-contact injuries accounted for a higher proportion of training injuries in senior compared to youth players (*P* = 0.002) (Table 3). The incidence and burden for cause of injury for the men’s senior and youth groups is shown in Table 4.

#### Illness

No significant difference occurred in reported illness incidence between senior and youth groups (0.58 vs. 0.59 illnesses per 1000 h) (*P* = 0.861) or illness burden (2.5 vs. 2.5 days absent per 1000) h (*P* = 0.714). The median severity as a result of an illness was 2 (senior) and 3 (youth) days.

### International Women’s Football

#### Exposure

For the women’s international squads, the total exposure over the duration of the study period was 80,766 h (senior: 29,616 h; youth: 51,150 h), consisting of 8026 match hours (senior: 3037 h; youth: 4989 h) and 72,740 training hours (senior: 26,579 h; youth: 46,161 h). There were 25 ± 1 (senior) and 22 ± 4 (youth) players involved in each camp and 15 ± 5 senior and 32 ± 8 youth camps per season; resulting in 129 ± 50 senior and 192 ± 57 youth camp days per season. There were 23 ± 8 senior and 39 ± 10 youth matches per season, resulting in 380 ± 135 (senior) and 624 ± 161 (youth) hours of match exposure per season. There were 3322 ± 1508 (senior) and 5770 ± 1346 (youth) hours of training exposure per season.

#### Injury Incidence

**Fig. 3:**
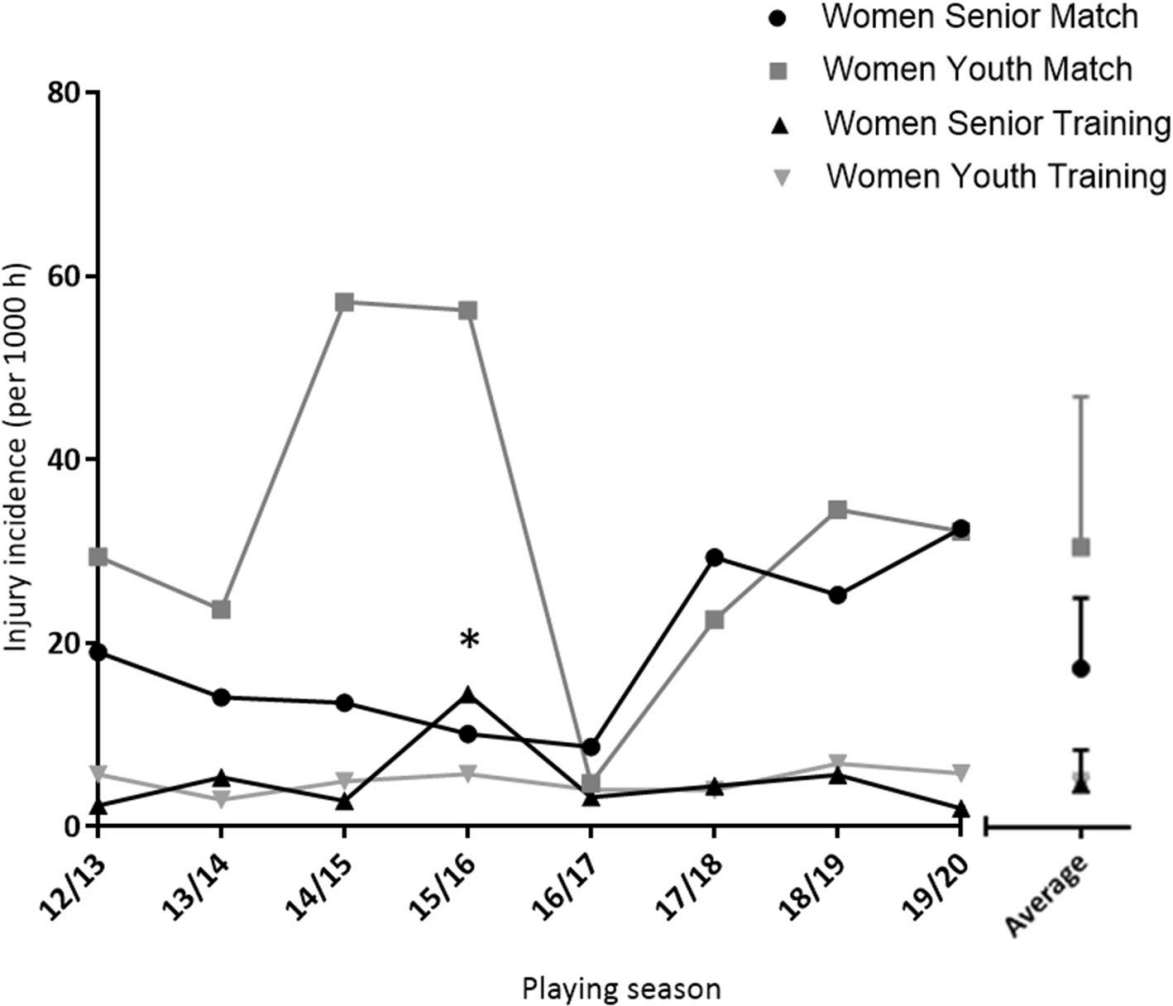
Injury incidence (per 1000 h) for senior and youth women’s international teams from 2012 to 2020. *Denotes a significant difference in training injury incidence between the senior and youth group for the 2015 to 16 season (*P* = 0.022)

There were a total of 503 injuries recorded (177 senior; 326 youth) across the 8 seasons, consisting of 191 match injuries (58 senior; 133 youth) and 312 training injuries (119 senior, 193 youth). Match injury incidence was 18.5 ± 8.2 (senior) and 32.6 ± 17.5 (youth) injuries per 1000 h, and training injury incidence was 5.0 ± 4.0 (senior) and 5.0 ± 1.3 (youth) injuries per 1000 h (Fig. 3).

Overall match injury incidence (27.6 ± 11.3 injuries per 1000 h) was higher in comparison to training injury incidence (5.1 ± 1.8 injuries per 1000 h) (main effect of event, *P* < 0.001), with no difference observed over 8 seasons (main effect of season, *P* = 0.296) and no event × season interaction (*P* = 0.578). Match injury incidence was higher than training injury incidence for the senior group (18.5 ± 8.2 vs. 5.0 ± 4.0 injuries per 1000 h; main effect of event, *P* < 0.001); however, there was no difference observed over 8 seasons (main effect of season, *P* = 0.617) and no event × season interaction (*P* = 0.337). Match injury incidence was also higher than training injury incidence for the youth group (32.6 ± 17.5 vs. 5.0 ± 1.3 injuries per 1000 h; main effect of group, *P* < 0.001); however, there was no difference observed over 8 seasons (main effect of season, *P* = 0.362) and no event × season interaction (*P* = 0.457).

There was no significant difference in match injury incidence between the senior and youth groups (main effect of group, *P* = 0.121), or over the course of 8 seasons (main effect of season, *P* = 0.758), with no group × season interaction (*P* = 0.707). There was no significant difference in training injury incidence between the senior and youth groups (main effect of group, *P* = 0.985). However, a significant difference was observed over the course of 8 seasons (main effect of season, *P* = 0.006), where training injury incidence was greater in 2015–2016 compared to 2013–2014 (*P* = 0.037), 2014–2015 (*P* = 0.023), 2016–2017 (*P* = 0.011), 2017–2018 (*P* = 0.033), and 2019–20 (*P* = 0.041). Additionally, a group × season interaction was observed (*P* = 0.021), where training injury incidence was greater in the senior group (14.4 injuries per 1000 h) compared to the youth group (5.7 injuries per 1000 h) during the 2015–2016 season alone (*P* = 0.022); training injury incidence was similar between senior and youth groups across the remaining 7 seasons (all *P* > 0.05).

#### Injury Burden

**Fig. 4:**
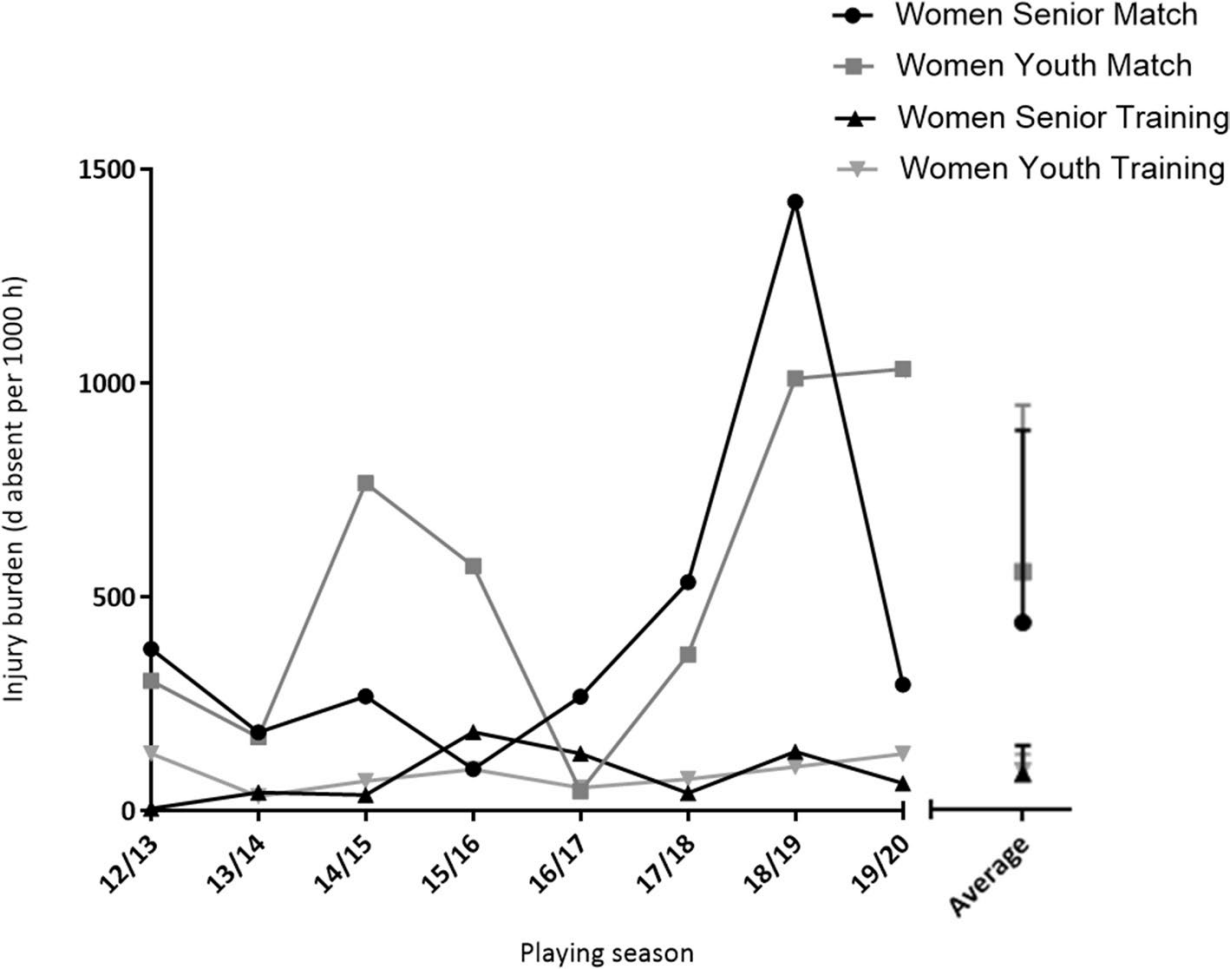
Injury burden (days absent per 1000 h) for senior and youth women’s international teams from 2012 to 2020

The match injury burden of women’s international football was 421.3 ± 430.5 (senior) and 534.8 ± 374.4 (youth) days absent per 1000 h, while the training injury burden was 82.0 ± 62.9 (senior) and 88.3 ± 36.0 (youth) days absent per 1000 h (Fig. 4).

Overall match injury burden (506.7 ± 350.2 days absent per 1000 h) was higher in comparison to training injury burden (87.6 ± 32.8 days absent per 1000 h) (main effect of event, *P* < 0.001), although no difference was observed over the 8 seasons (main effect of season, *P* = 0.060), and no event × season interaction (*P* = 0.127). There was no difference in match and training injury burden for the senior group (421.3 ± 430.5 vs. 82.0 ± 62.9 days absent per 1000 h; main effect of group, *P* = 0.076), or over the course of 8 seasons (main effect of season, *P* = 0.438), with no event × season interaction (*P* = 0.527). Match injury burden was higher than training injury burden for the youth group (534.8 ± 374.4 vs. 88.3 ± 36.0 days absent per 1000 h; main effect of group, *P* = 0.001); however, there was no difference observed over 8 seasons (main effect of season, *P* = 0.231) and no event × season interaction (*P* = 0.374).

There was no difference in match or training injury burden between the senior and youth groups (main effect of group: match: *P* = 0.603; training: *P* = 0.802), or over the course of 8 seasons (main effect of season: match: *P* = 0.182; training: *P* = 0.395), with no group × season interaction (match: *P* = 0.807; training: *P* = 0.419).

#### Injury Severity

There was no difference in the distribution of injuries between match-play and training (*P* = 0.064). There was no difference in the distribution of injuries between senior and youth groups for overall injury severity (*P* = 0.387) and training injury severity (*P* = 0.596). However, for match injury severity, the distribution of minor injuries was greater in the youth group (*P* = 0.002), whereas the distribution of moderate and major injuries was greater in the senior group (*P* = 0.002), with no difference in the distribution of severe injuries (Table 5). The incidence for severity of injury for the women’s senior and youth groups is shown in Table 6.

**Table 5:** Injury severity, cause, and onset in women’s senior and youth groups [injury number and proportion (%)]

	Both groups			Senior			Youth		
	All injuries	Match injuries	Training injuries	All injuries	Match injuries	Training injuries	All injuries	Match injuries	Training injuries
Severity	(*n* = 500)	(*n* = 191)	(*n* = 309)	(*n* = 177)	(*n* = 58)	(*n* = 119)	(*n* = 323)	(*n* = 133)	(*n* = 190)
Minor	247 (49%)	93 (49%)	154 (50%)	81 (46%)	17 (29%)	64 (54%)	166 (51%)	76 (57%)^a^	90 (47%)
Moderate	172 (34%)	60 (31%)	112 (36%)	68 (38%)	28 (48%)^b^	40 (34%)	104 (32%)	32 (24%)	72 (38%)
Severe	63 (13%)	26 (14%)	37 (12%)	20 (11%)	8 (14%)	12 (10%)	43 (13%)	18 (14%)	25 (13%)
Major	18 (4%)	12 (6%)	6 (2%)	8 (5%)	5 (9%)^b^	3 (3%)	10 (3%)	7 (5%)	3 (2%)
Cause	(*n* = 381)	(*n* = 150)	(*n* = 231)	(*n* = 120)	(*n* = 40)	(n = 80)	(*n* = 261)	(*n* = 110)	(*n* = 151)
Contact	152 (40%)	82 (55%)^c^	70 (30%)	42 (35%)	17 (43%)	25 (31%)	110 (42%)	65 (59%)	45 (30%)
Non-contact	180 (47%)	51 (34%)	129 (56%)^d^	59 (49%)	16 (40%)	43 (54%)	121 (46%)	35 (32%)	86 (57%)
Cumulative	49 (13%)	17 (11%)	32 (14%)^d^	19 (16%)	7 (18%)	12 (15%)	30 (12%)	10 (9%)	20 (13%)
Onset	(*n* = 445)	(*n* = 168)	(*n* = 277)	(*n* = 151)	(*n* = 46)	(*n* = 105)	(*n* = 294)	(*n* = 122)	(*n* = 172)
Acute	356 (80%)	142 (85%)	214 (77%)	121 (80%)	39 (85%)	82 (78%)	235 (80%)	103 (84%)	132 (77%)
Gradual	89 (20%)	26 (15%)	63 (23%)	30 (20%)	7 (15%)	23 (22%)	59 (20%)	19 (16%)	40 (23%)

NB The *n* represents the number of injuries for which the severity, cause, and onset were known in each category (3 injuries had an unknown severity, 122 injuries had an unknown cause, and 58 injuries had an unknown onset)

^a^Greater proportion in youth compared to senior

^b^Greater proportion in senior compared to youth

^c^Greater proportion in match compared to training

^d^Greater proportion in training compared to match

**Table 6:** Injury incidence (injuries per 1000 h) and burden (days absent per 1000 h) severity, cause, and onset in women’s senior and youth groups

	Both groups						Senior						Youth					
	All injuries		Match injuries		Training injuries		All injuries		Match injuries		Training injuries		All injuries		Match injuries		Training injuries	
	Incidence	Burden	Incidence	Burden	Incidence	Burden	Incidence	Burden	Incidence	Burden	Incidence	Burden	Incidence	Burden	Incidence	Burden	Incidence	Burden
Severity																		
Minor	3.1	11.2	11.6	38.9	2.1	8.2	2.7	10.3	5.6	18.1	2.4	9.4	3.2	11.7	15.2	51.5	1.9	7.5
Moderate	2.1	32.5	7.4	111.6	1.5	23.7	2.3	35.2	8.9	146.2	1.5	22.5	2.0	30.9	6.4	90.6	1.6	24.4
Severe	0.8	38.2	3.2	163.9	0.5	24.4	0.7	33.2	2.6	119.9	0.5	23.3	0.8	41.2	3.6	190.6	0.5	25.0
Major	0.2	35.8	1.5	239.5	0.1	13.3	0.3	39.0	1.6	253.9	0.1	14.4	0.2	33.9	1.4	230.7	0.1	12.6
Cause																		
Contact	1.9	35.4	10.2	252.1	1.0	11.5	1.4	22.5	5.6	156.4	0.9	7.2	2.2	42.9	13.0	310.3	1.0	14.0
Non-contact	2.2	42.2	6.2	120.4	1.8	33.5	2.0	41.6	4.9	111.6	1.6	33.6	2.4	42.5	7.0	125.7	1.9	33.5
Cumulative	0.6	8.7	2.1	54.6	0.4	3.6	0.6	7.4	2.3	44.8	0.5	3.1	0.6	9.4	2.0	60.5	0.4	3.9
Onset																		
Acute	4.4	84.3	17.6	412.1	2.9	48.2	4.1	72.0	12.5	290.8	3.1	47.0	4.6	91.5	20.6	485.9	2.9	48.9
Gradual	1.1	15.1	3.2	46.6	0.9	11.6	1.0	12.6	2.3	51.0	0.9	8.2	1.2	16.5	3.8	43.9	0.9	13.6

N.B. 1 match injury was removed from senior women analyses where required as exposure was not received

#### Onset of Injury

There was no difference in the distribution of injuries between match-play and training (*P* = 0.063). There was no difference in the distribution of injuries between senior and youth groups for the overall onset of injury (*P* = 0.960), onset of injuries in matches (*P* = 0.955), and the onset of injuries in training (*P* = 0.795) (Table 5). The incidence and burden for onset of injury for the women’s senior and youth groups is shown in Table 6.

#### Cause of Injury

The distribution of contact injuries was greater in match-play compared to training (*P* < 0.001), while non-contact and cumulative injuries accounted for more training injuries compared to match-play (*P* < 0.001). There was no difference in the distribution of injuries between senior and youth groups for the cause of injury in match-play and training combined (*P* = 0.302), in match-play alone (*P* = 0.143), or in training alone (*P* = 0.883) (Table 5). The incidence and burden for cause of injury for the women’s senior and youth groups is shown in Table 6.

#### Illness

There were 59 illnesses (21 senior, 38 youth) reported during the study. No significant difference in illness incidence was observed between senior and youth groups (0.71 vs. 0.74 illnesses per 1000 player h; main effect of group, *P* = 0.993) or illness burden (2.8 vs. 3.8 days absent per 1000 player h; main effect of group, *P* = 0.905). The median days lost as a result of an illness were 2 (senior) and 3 (youth).

## Discussion

The main findings of the present study indicate that male and female senior and youth international football players are exposed to a high risk of injury, especially during matches. In both men’s and women’s international football, match injury incidence was higher than training injury incidence (men’s: 31.1 vs. 4.0; women’s: 27.6 vs. 5.1; injuries per 1000 h respectively) and match injury burden was higher than training injury burden (men’s: 454.0 vs. 51.0 days; women’s: 506.7 vs. 87.6; days absent per 1000 h, respectively). In men’s and women’s international football, there was no difference in injury incidence or burden between the senior and youth groups. In men’s international football, match injury incidence was shown to be higher in the senior group for the 2012–2013 season compared to 2019–2020, which is potentially explained by the truncated data in the 2019–2020 season due to the outbreak of the coronavirus pandemic (March 2020). In women’s international football, training injury incidence was greater in the senior group compared to the youth group during the 2015–2016 season.

### Incidence of Injuries in Men’s International Football

#### Match Injury Incidence

Match injury incidence in men’s international football in the present study was 31.8 (senior) and 29.8 (youth) injuries per 1000 h of match-play. Time-loss match injury incidence in the present study is lower than data derived from 5 World Cup tournaments (1998–2014) (44.7 injuries per 1000 h [38]) and a meta-analysis of men’s international football consisting of 13 studies reporting match injuries (41.1 injuries per 1000 h; [15]). The reason for the match injury incidence being lower in the present study may be due to the majority of studies assessing match injury incidence in men’s international football focusing exclusively on tournament settings for their period of data collection [38–41]. While match injury incidence during tournament football is of interest, this covers a relatively small percentage of the international football calendar. By providing data on all international football (friendlies, qualifying matches, and tournaments), the present study provides a more accurate representation of match injury incidence across all international football match activity. It could be suggested that match injury incidence is higher in tournament-based football due to the greater perceived importance of tournament football over friendly and qualifying matches. Thus, coaches may employ less squad rotation, possibly due to a smaller, fixed, squad size; leading to players playing numerous matches in a short space of time [5, 6]. Also, as tournament match-play could be deemed more competitive than friendly or qualifying fixtures and teams are potentially more evenly matched in terms of playing ability, possession is likely to be exchanged more often and, as a result, could lead to greater injury risk [42].

It has previously been speculated that match injury incidence may differ between men’s international and club football due to the different context and possibly unaccustomed style of play that may be encountered in international football. However, match injury incidence in the current study is comparable to the match injury incidence of clubs participating in the Champions League (30.5 injuries per 1000 h; [43]), Spanish Liga 1 (43.5 injuries per 1000 h; [44]), UEFA elite clubs (26.6 injuries per 1000 h, [7]), Dutch Eredivisie (32.8 injuries per 1000 h; [45]), and data from a meta-analysis of club football consisting of 27 studies (32.3 injuries per 1000 h; [15]). The similarities may be due to the comparable demands, in terms of intensity and match-play characteristics, and the multitude of contextual factors that are shared by international and club football [46]. The present study however extends these findings and provides insight into the match injury incidence across 8 seasons of men’s international football.

#### Training Injury Incidence

Training injury incidence in men’s international football in the present study was 3.8 (senior) and 4.0 (youth) injuries per 1000 h of training. There is a lack of research assessing training injury incidence in men’s international football in the last decade, outside of a tournament setting. Training injury incidence observed in a meta-analysis of 6 studies from men’s international football was 3.5 injuries per 1000 h [15]. However, of the 6 studies assessed in the meta-analysis, 4 studies only assessed training injury incidence during a tournament (World Cup, [39]; European Championships U19–Senior, [12, 40]; Gulf and Asian Cup, [41]). While these data are of interest, training taking place during a tournament can be very different to training that takes place during international camps due to the shorter length of a training camp in comparison to a tournament (typical training camp: ~ 10 days for friendly and qualifying matches, ~ 33 days for a tournament). The shorter camp lengths make periodisation of training more challenging and, therefore, could affect subsequent injury incidence [47]. The only study to examine international football across tournaments, qualifying fixtures and training camps in the last 10 years, assessed the Qatari national senior team [48]. A training injury incidence of 4.3 injuries per 1000 h was shown; however, the relatively short nature of the study compared to the current study (17-months vs 96-months) and the difference in playing standard (Qatar ranking October 2008: 78th vs. current (2020) England ranking: 4th) questions whether the previous study is a true reflection of training injury incidence in elite international football.

Training injury incidence in the current study aligns with research in club football. A meta-analysis of 27 studies assessing training injury in club football reported 3.8 injuries per 1000 training hours in professional players [15]. These findings suggest that training injury incidence does not differ between club football and international football. It has previously been suggested that a player training for their national team could be at a heightened risk of injury, possibly due to the increased risk that unaccustomed training represents [4]. However, it may be that the greater ability to objectively monitor player training loads [49] and communication between club and international team regarding individual players’ fitness status negate the proposed additional injury risk posed by participation in international football.

### Incidence of Injuries in Women’s International Football

#### Match Injury Incidence

In the first study of its kind in women’s international football, the present study reports a match injury incidence of 18.5 injuries per 1000 h for senior players. Previous studies involving international women’s players have focused only on tournament-based match-play [28, 29]. During women’s international tournaments staged between 1999 and 2006, involving U19-Senior players, the match injury incidence ranged between 20 and 49 injuries per 1000 h [28]. In FIFA World cups and Olympic games staged between 1999 and 2011, involving U17-Senior players, time-loss injuries ranged from 0.7 to 1 per match [29]. The injury incidence in the current study is at the lower end of the previously reported range. This could be due to previous studies focusing on tournament match-play [28, 29], which inherently involves a congested fixture schedule, resulting in an increased susceptibility to injury [5, 6]. The large range in injury incidence in previous studies may reflect the relatively low number of games played in some international tournaments (≤ 32 matches) compared to 183 matches (senior) in the current study. The lower number of games could lead to the injury incidence metrics being more sensitive to clustered or unusual injury occurrences. The incidence of injury in women’s club football has been reported to be 16.1 (League: Damallsvenskan, Year of data collection: 2005, [24]), 13.9 (Damallsvenskan [25]), 22.6 (Primera Iberdrola, 2010–2015, [26]), and 12.6 (USA National Women's Soccer League, 2001–2002, [27]) injuries per 1000 h of match-play. These data seem to align with the incidence of injury in senior international football. This may be due to the demands of playing international football being similar to playing elite football in the top European and North America leagues. However, a detailed study examining the demands of elite female football is required to substantiate or refute this suggestion.

The incidence of injury in youth female international players is not well established as very little published data exist documenting injury in this population. In the current study, match injury incidence was 32.6 injuries per 1000 h in elite youth players over the course of 8 seasons. The only other study to document such information in elite female youth players observed a match injury incidence of 22.4 injuries per 1000 h in players based at the Clairefontaine elite academy in France [50]. The reason for the greater incidence of injury in the present study could be due to the current data being collected more recently. The present study is based on data collected between the 12/13 and 19/20 season, while the study by Le Gall and colleagues [50] is based on data collected between 1998 and 2006. It is well established that the women’s game has grown exponentially between 1998 and 2018 [17] and the demands of the game are also likely to have increased in this time [51]. The present study, for the first time, documents the impact of these changes on match injury incidence in elite female youth football players.

#### Training Injury Incidence

In women’s international football, the incidence of injuries occurring as a result of training was 5.0 injuries per 1000 h for both the senior and youth groups. In the women’s international senior championships in 2005, the injury incidence was 2.3 injuries per 1000 h of training [40]. The higher injury incidence in the present study may be due to the training in tournament football being of a less intense nature compared to other international camps due to the fixture congestion associated with tournament-based football [52]. This may have led to training tending to focus more on recovery, which, due to the reduced intensity, may carry less of an injury risk [53]. In elite club football, the incidence of training injury has been shown to also be slightly lower than in the present study (1.2–3.8 injuries per 1000 h of training activity; [24–27]). The higher training injury incidence in international football may be due to the unaccustomed nature of international football in comparison to club football, which could lead to different type and magnitudes of musculoskeletal strain being required, thus presenting a greater risk of injury [54]. Other factors that could be suggested to have caused an increase in training injury incidence include players experiencing varying degrees of chronic loading before being selected to represent their international team, increased competitiveness for international match selection, and an increased intensity of training, particularly in youth players. Further research in women’s international football is however required to explore these suggestions.

### Burden of Injuries in Men’s International Football

The present study provides novel evidence that the injury burden of men’s international football is 455.7 (senior) and 450.0 (youth) days absent per 1000 match hours and 36.2 (senior) and 60.2 (youth) days absent per 1000 training hours. There is a lack of published data in men’s international football assessing the injury burden. This is a concern due to injury burden arguably being more important than injury incidence for medical practitioners and subsequent player availability [55].

In men’s club football, injury burden was reported as 130 days absent per 1000 player hours [7], compared to 98 (senior) and 122 (youth) days absent per 1000 player hours in the present study. The reason for men’s international football training having a slightly lower injury burden in comparison to club football could be due to several factors. Medical practitioners in international football may adopt a more conservative approach to whether a player is withdrawn from activity when suffering minor discomfort, particularly in friendly matches, due to the perceived importance of club football over these types of international fixtures. Also, clubs are potentially less likely to encourage their players to attend international camps when returning from injury, thus decreasing injury risk [56]. While these data suggest that the injury burden of men’s international football is lower that club football, the lack of distinction between injuries sustained in training and match-play when assessing burden in club football leads to unanswered questions about the comparative burden between club and international training and match-play. This warrants further investigation given the importance of understanding injury burden in applied practice.

### Burden of Injuries in Women’s International Football

The present study provides novel evidence that the injury burden associated with women’s international football is 421.3 (senior) and 534.8 (youth) days absent per 1000 match hours and 82.0 (senior) and 88.3 (youth) days absent per 1000 training hours. Injury burden is not commonly reported in women’s international football, with a large proportion of studies focusing on the incidence of injuries. In the absence of any comparable data in female international football, injury burden in men’s international football can be used as a benchmark. In the present study, injury burden in women is higher than in their male equivalents (match injury burden in men’s international football = 454.0 days absent per 1000 match h; training injury burden in men’s international football = 51.0 days absent per 1000 training h). Exploration of the reasons why injury burden appears greater in women’s compared with men’s international football is beyond the scope of the current study, but future research should investigate injury burden in women’s international football to enhance injury surveillance and to inform the development of appropriate mitigation procedures.

### Severity and Onset of Injuries in Men’s International Football

#### Severity

The percentage of minor injuries sustained in men’s international football was 60% (senior) and 59% (youth), while the percentage of minor injuries reported in the previous studies cover a wide range (49–67%) [12, 39, 48]. The reason for the differing reported percentages in previous studies may be due to the single tournament setting of some studies [39] leading to a relatively small number of injuries being reported. The lack of longitudinal studies in men’s international football mean that the number of severe and major injuries reported is low or not reported [12, 39, 48], potentially due to the difficultly in following up on teams after the tournament was completed. An advantage of the present study is that players’ clubs were contacted to follow up on long-term injuries that were rehabilitated after the international window. A lack of follow-up in previous studies may cause erroneous assumptions to be made regarding the severity of injury in international football. In club football, where the follow-up is likely to be more accurate, the percentage of minor (49–62.5%), moderate (29.2–34.3%), and severe/major injuries (8–15%) [44, 45] is similar to the distribution in the present study.

#### Onset of Injury

Acute onset of injury was the most common in the men’s senior (80%) and youth (73%) groups. In a tournament setting, the percentage of injuries that occur acutely has been previously reported as 84% [40] and 77% [12], while in men’s international football in general, the values are comparable (80%) [56]. In men’s club football, the percentage of injuries that have an acute onset is lower than in international football (69% Dutch league [44]; 35% Spanish league [45]). A potential reason for the relative lack of gradual onset injuries in international football could be that players are not selected for their international team if they are experiencing the early symptoms of what could be a gradual onset injury. The onset of injury is however difficult to compare between studies, due to the difficultly in confirming injury onset; in some cases, the onset could be a combination of acute and gradual and exist on a continuum [36]. This subjective nature of categorisation could lead to a difference of opinion amongst practitioners, which could be further exacerbated in club football where medical teams mainly work independently of each other. The present study is however the first to accurately depict the severity and onset of injuries in men’s international football, in senior and youth age groups, across 8 seasons of competition.

### Severity and Onset of Injuries in Women’s International Football

#### Severity

The present study showed minor injuries to be the most common injury severity in women’s international football (youth: 51%; senior: 46%), followed by moderate (youth: 32%; senior: 38%), severe (youth: 13%; senior: 11%), and major injuries (youth: 3%; senior: 5%). Research incorporating the overall severity of injuries at an international level in women’s football is scarce, possibly due to the lack of monitoring of players outside of a tournament setting. The present findings align with women’s club football reporting minor/mild injuries (38%–53%) as the most common, followed by moderate (34–40%) and severe/major injuries (12–23%) [24, 26]. The results of the present study fall within the range of values previously reported in the published literature for each injury severity. However, it should be noted that the ranges are large and may be a result of different playing abilities across numerous populations in different leagues.

#### Onset of injury

Acute-onset injuries were the most common in senior (overall: 80%; match: 85%; training: 78%) and youth (overall: 80%; match: 84%; training: 77%) women’s international players in the present study. During international tournament football, it was shown that 83% of injuries were caused acutely [40], with similar findings observed in elite youth football (86%, [50]). Additionally, the majority of injuries in women’s club football are as a result of acute incidents (51–90%); [26, 27]. The onset of acute injuries appears to be consistent across all levels of women’s football. An explanation for this could be due to the actions that are generally associated with acute injuries in football training and match-play (i.e., kicking, heading, and tackling) are similar across domestic and international competition.

In the present study, contact injuries accounted for 40% of all injuries (senior: 35%; youth: 42%). In international football tournaments, the majority of injuries were also reported as contact (84%—[28]; 83% senior world cups, 87% U19/U20 world cups, and 75% U17 world cups, [29]). The lower percentage of contact injuries in the present study may be due to the longitudinal nature of the current study and the possible difference in context between tournament football and friendly and qualifying fixtures [46]. The greater amount of match exposure in a short time-period within tournament football coupled with the competitiveness of tournament match-play may also have caused an increase in contact injuries. Tournament football is associated with high pressure for international teams, potentially increasing the competitiveness and physicality of match-play. This may lead to an increase in tackles, collisions, and aerial duels, thus increasing the risk of contact injuries occurring. The majority of injuries in women’s club football are as a result of non-contact incidents (74%, Swedish—2005—[24]; 81%, Spanish—2010–2015—[26]). However, there is a lack of consistency in the reporting of injury causation in women’s football. Guidelines on the collection and reporting of injury surveillance information [36] need to be adhered to before consensuses can be made and preventative strategies can be employed. The present study does however, for the first time, provide an insight into the severity and onset of injuries across 8 seasons of women’s international football.

### Senior vs. Youth Age Group Comparisons

#### Men’s International Football

There was no difference in the match and training injury incidence and burden between senior and youth men’s international teams. It has previously been reported that youth footballers have an increased incidence of injury in comparison to adults [58] and the likelihood of injury increases through the youth age groups in men’s football [58]. The reason for the increase in injury incidence has been attributed to the increased competitiveness of youth football as a footballer progresses through the advancing age groups [30]. However, previous studies focused on players of varying abilities within club football, based at numerous different clubs [15], with assumed different styles and intensity of play. This is an important consideration given that lower football ability/skill-level has been shown to increase injury risk [57, 59], possibly due to less technical ability when tackling or being tackled and/or an inability to cope with the physical demands that football necessitates. As the present study was conducted in international players, it is assumed that the players are highly skilled and, therefore, any injury risk attributed to poor technique or lack of physical ability is reduced.

The present study found no difference in the distribution of injuries between the senior and youth groups regarding injury severity and the onset of injury. However, a difference was shown for the cause of injury, where the youth group obtained a higher distribution of contact injuries compared to the senior group overall, and in training alone. This may be due to less experience and lower technical ability of youth footballers compared to their senior counterparts [57], leading to inadvertent contact taking place. In summary, the present study provides novel evidence regarding the epidemiology of injury and illness in international men’s football, and documents no differences between youth and senior player’s injury incidence and burden at this very highest level of competition, whilst youth players experience a higher proportion of contact injuries.

#### Women’s International Football

In women’s international football, there was no difference in match or training injury incidence observed between senior and youth teams [match injury incidence: 18.5 (senior), 32.6 (youth) injuries per 1000 h; training injury incidence: 5.0 (senior), and 5.0 (youth) injuries per 1000 h]. However, during the 2015–2016 season, training injury incidence was higher in the senior group (14.4 injuries per 1000 h) compared to the youth group (5.7 injuries per 1000 h). The reason for the difference in training injury incidence between senior and youth groups for the 2015–2016 season could be due to the increased professionalism of the women’s game and World Cup commitments. In 2011, the Women’s Super League (WSL) consisted of only 8 teams; this has increased by 50% to 12 teams in 2020. An increase in the professionalisation of women’s football in 2015 resulted in an increase in full-time training at club level, which would have led to a large increase in overall training exposure, and thus potentially increased injury incidence.

Although match injury incidence was 44 and 46 injuries per 1000 match hours greater in the youth group compared to the senior group in the 2014–2015 and 2015–2016, no statistically significant difference was shown (Fig. 3). Similarly, no differences in match or training injury burden were observed between senior and youth international teams. However, in the 2018–2019 season, 1013 (senior) and 477 (youth) more days were lost per 1,000 match hours compared to the average injury burden over the course of the 8-season study. The reason for the lack of statistically significant differences may be due to the large variability in inter-seasonal injury incidence and burden, exemplified by the large standard deviations shown. Fluctuations could be a result of many factors, including but not limited to: playing style, opposition, personnel selected, squad size, and variation in the number of matches per season*.* Despite the aforementioned postulations, it is unclear why fluctuations in injury incidence and burden occurred in the present study. The assessment of injury types, locations and patterns in future research may provide a greater insight into the factors that contribute to injury incidence and burden.

The present study found no difference in the distribution of injuries between the senior and youth groups regarding onset and cause of injury in women’s international football. The lack of a difference between the senior and youth groups may also be due to the preparation, philosophies, and processes being the same throughout the international football age groups. However, a difference was shown in match injury severity, where the youth group obtained a higher distribution of minor injuries compared to the senior group. This may be a result of practitioners and coaches taking greater precaution with youth players, as well as younger players having less technical experience and ability compared to senior players, potentially leading to an increase in late or technically improper challenges causing more minor injuries (e.g. haematomas).

### Illness

#### Illness in Men’s International Football

Incidence of illnesses was 0.58 (senior) and 0.59 (youth) per 1000 player hours with a burden of 2.5 (senior) and 2.5 (youth) days absent per 1000 player hours in men’s international football. In a study of 73 men’s professional clubs, across 4 seasons, it was reported that 1.5 illnesses occurred per 1000 player days [60]. However, illnesses vary in the timescale it takes before they become symptomatic; therefore, it is difficult to ascertain if an individual became infected when representing their club or national team. As a result of this, caution should be taken when comparing illness incidence between international and club football.

#### Illness in Women’s International Football

Illnesses occurred at an incidence of 0.71 (senior) and 0.74 (youth) per 1000 player hours, with a burden of 2.8 (senior) and 3.8 (youth) days absent per 1000 player hours in women’s international football. The median severity as a result of an illness was 3 (senior) and 2 (youth) days absent. There is a scarcity of research examining the epidemiology and influence of illness in women’s international football. Due to the relatively low incidence and burden of illnesses in women’s international football, there is a potential for them to be seen as insignificant and not of great concern to practitioners. However, it is likely that some players continue to participate in training and match-play when suffering from an illness, which may lead to sub-optimal performance. Greater reporting of illnesses and how they may influence playing performance is required before the influence of illness on women’s international football is truly elucidated.

### Limitations

Although this is the first paper to conduct a study of this nature in international football, it is not without limitation. It is acknowledged that the intensity of match-play and training characteristics may influence injury and illness epidemiology. However, due to the size of the study and the number of players involved, information on players’ training and match-play characteristics (i.e., workload variables such as total distance covered and high-speed running distance) that may contribute to the onset and nature of injuries and illnesses was not reported. It is also acknowledged that injuries and illnesses are influenced by a variety of factors, including training history. Players involved in the present study were also training and playing for their club teams outside of international commitments. Players’ chronic workload and prior training status was not monitored, and therefore, these factors could have influenced injury and illness susceptibility. It is also acknowledged that although all players were treated for their injuries by medical professionals, different practitioners have been utilised between squads and over the duration of the study, leading to different approaches to rehabilitation and, subsequently, this may have influenced player recovery time, and subsequent re-injury risk. However, this is impossible to avoid in large-scale studies of this nature. Furthermore, it must be noted that the 2019/20 season was truncated by the outbreak of the coronavirus pandemic (March 2020) and, therefore, may not be a true representation of an international season. A comparison between the epidemiology of injury and illness men and women may be of interest; however, due to the established anthropometric and physiological differences between men and women footballers [33, 34], an attempt to make sex-specific comparisons was not made. Furthermore, these comparisons were not the aim of the current study and, therefore, fall outside of the scope of this paper.

## Conclusion

The findings of the present study provide a holistic and comprehensive examination of injury and illness epidemiology in men’s and women’s international football squads (U15—Senior) across 8 seasons of match-play and training. The present study enhances the knowledge of injury and illness in an elite international setting in senior and youth groups, and provides essential guidance to practitioners regarding match and training injury epidemiology. Practitioners will be able to accurately benchmark their team’s injury and illness characteristics to the match-play and training information provided in present study.

## Data Availability

Limited, anonymised data will be made available where appropriate via contact with the corresponding author.
