# Correction to: The Football Association Injury and Illness Surveillance Study: The Incidence, Burden and Severity of Injuries and Illness in Men’s and Women’s International Football

**DOI:** 10.1007/s40279-023-01971-5

**Published:** 2023-11-24

**Authors:** Bradley Sprouse, Jon Alty, Steve Kemp, Charlotte Cowie, Ritan Mehta, Alicia Tang, John Morris, Simon Cooper, Ian Varley

**Affiliations:** 1 Sport Science Department, Nottingham Trent University, Nottingham, UK; 2 The Football Association, Burton-Upon-Trent, UK

**Correction to: Sports Medicine** 10.1007/s40279-020-01411-8

Following online publication, concerns were raised regarding presentation of the study’s methodology, data collection and analysis. The reported study represents the retrospective analysis of a set of data that was collected as part of a wider injury and illness surveillance programme (IISP). The retrospective nature of the analysis was not made expressly clear in the original publication, and the following corrections should be noted: 

**Section 2.2 Participants**

The second paragraph of this section refers to ethics approval granted by the Nottingham Trent University Non-Invasive Ethical Review Committee. The ethics approval granted by the Nottingham Trent University Non-Invasive Ethical Review Committee related to the retrospective analysis of previously collected data only. It does not relate to data collection.

**Section 2.3 Data Collection**

The first paragraph of this section refers to data collection and the recording of injury severity.

Injury severity data were logged based on the number of days that players were unavailable for training and/or match-play. It should be clarified that categorisation of injury severity did not take place at the time of data collection. BS and IV retrospectively categorised the raw data used within the study into minor, moderate, severe, and major categories based on the number of days absent.

The fourth paragraph of this section refers to guidance documents. It should be noted that these documents included the following:

*Fuller* *and* *Taylor* *(2013).* *The* *Football* *Association* *Injury* *and* *Illness* *Surveillance* *Study:* *Guidance* *Document.*

**Acknowledgements**

Written permission to be named in this specific article was not obtained from those originally included in the Acknowledgements section. This section has since been removed.

**Author Contributions**

The Author Contributions statement includes the following statement:

*Material preparation, data collection, and analysis were performed by IV, BS, JA, and SK.*

It should be noted that data were collected by the coaches and FA-appointed medical staff caring for each the relevant teams. As well as collecting data themselves, SK and JA provided oversight of staff to ensure data were collected appropriately for the injury and illness surveillance project. BS, IV, SK and JA were involved in the preparation of material used for some data collection, and BS and IV conducted the analysis.

